# Short-term HRV changes across gaming and simulation modalities with varying levels of immersion and physical demand

**DOI:** 10.3389/fpsyg.2026.1829885

**Published:** 2026-05-20

**Authors:** Aylin Zekioglu, Nihal Dal, Serdar Tok, Naci Kalkan, Said Enes Yilmaz, Erdal Binboga, Ilker Balikçi

**Affiliations:** 1 Faculty of Sports Sciences, Manisa Celal Bayar University, Manisa, Türkiye; 2 Department of Biophysics, Faculty of Medicine, Ege University, Izmir, Türkiye

**Keywords:** autonomic nervous system, gaming, heart rate variability, immersion, simulation, virtual reality

## Abstract

Virtual reality (VR), gaming, and simulation technologies are increasingly used for training, skill development, and rehabilitation. However, there is limited understanding of how such platforms may trigger autonomic nervous system (ANS) responses beyond baseline levels. It is also unclear whether varying levels of immersion, together with physical demand, influence these responses. This study aimed to investigate how different levels of immersion and physical demand across gaming and simulation modalities affect ANS responses, as measured by heart rate (HR) and heart rate variability (HRV), relative to baseline values. Eighty-seven undergraduate students (mean age = 20.5 years) were allocated to three experimental groups based on immersion level and task demand: immersive and high physical demand (VR table tennis, *n* = 17), semi-immersive and intermediate physical demand (iRacing car simulation, *n* = 40), and non-immersive and low physical demand (FIFA console soccer, *n* = 30). Participants completed a 4-min activity phase, preceded by a 4-min baseline and followed by a 4-min recovery period. HR and HRV were continuously recorded via ECG. Repeated-measures ANOVAs were used to examine differences across experimental periods, and one-way ANOVAs were conducted to compare percentage changes relative to baseline between groups. All three modalities led to a significant increase in HR and a reduction in vagally mediated HRV from baseline to activity, as evidenced by decreases in RMSSD and HF power. The subsequent recovery phase generally showed a return toward baseline values. Moreover, the VR Table Tennis group, representing the highest physical demand, demonstrated a significantly greater increase in HR and decrease in NNmean than both iRacing and FIFA during activity. These findings suggest that gaming and simulation modalities, regardless of immersion level, may elicit ANS responses above resting levels. The magnitude of the response appears to be more strongly influenced by physical demand than immersion alone.

## 1 Introduction

The processes of learning, training, and skill development within specialized fields such as athletics, the military, aviation, and surgery may involve substantial risk, cost, and logistical constraints. In addition, regulatory bodies often limit testing and training opportunities, particularly in domains such as motorsports. When access to facilities, equipment, or competitive environments is restricted because of injury, safety concerns, scheduling demands, or operational limitations, virtual systems can provide controlled and repeatable settings for practice and evaluation. In this context, gaming and simulation applications, including immersive and non-immersive formats, may offer practical alternatives. Previous research has suggested that virtual environments may support skill acquisition, perceptual training, and decision-making in both athletic and professional populations (Engelbrecht et al., 2019). In addition, many gaming and simulation tasks require rapid decision-making, visuomotor coordination, sustained attention, timing precision, and adaptive responses under time pressure. These features make them useful controlled models for investigating short-term psychophysiological demands.

Despite the potential of VR systems for skill acquisition, development, and training, it remains unclear whether these technologies evoke autonomic nervous system (ANS) responses beyond resting levels in ways relevant to task performance. This issue is important because the practical value of simulation systems may depend not only on visual realism but also on their ability to generate meaningful physiological and attentional demands comparable to real-world settings.

A further issue requiring investigation concerns the relationship between immersion level and autonomic responses. Immersion is commonly defined as the extent to which a system creates a sense of presence within a mediated environment (Freina and Ott, 2015). Virtual systems are generally classified as fully immersive, semi-immersive, and non-immersive formats (Cipresso et al., 2018), each differing in sensory input, user interaction, and movement demands.

Based on this framework, the present study examined three gaming modalities representing distinct combinations of immersion and task demand: fully immersive VR table tennis, semi-immersive iRacing simulation, and non-immersive FIFA console gaming. These tasks differ not only in display format, but also in movement requirements, perceptual processing, and likely psychophysiological load.

HR and HRV are widely used non-invasive indicators of short-term autonomic regulation during task engagement and recovery (Task Force of the European Society of Cardiology the North American Society of Pacing (Task Force of the European Society of Cardiology the North American Society of Pacing Electrophysiology, 1996; Laborde et al., 2017). Acute increases in HR together with reductions in vagally mediated HRV commonly reflect heightened task demand, effort mobilization, or movement-related physiological load (Aubert et al., 2003; Thayer and Lane, 2000). Accordingly, HRV may provide a practical marker for determining whether esports and simulation tasks generate demands above resting baseline levels. Recent evidence also supports HRV reactivity as a sensitive marker of acute psychophysiological adaptation and monitoring across diverse task environments (Manser et al., 2021).

Previous research examining virtual environments and HRV has focused primarily on aerobic exercise modalities such as cycling or moderate-intensity physical activity (Rutkowski et al., 2021; Ahmed et al., 2020). Comparatively little is known about short-duration precision- and skill-based tasks such as virtual table tennis, racing simulation, and football gaming. Moreover, fully immersive and non-immersive systems have generally been studied separately rather than within the same comparative framework.

Accordingly, the purpose of the present study was to compare short-term HR and HRV responses across three gaming modalities representing different combinations of immersion and physical demand: (1) immersive VR table tennis, (2) semi-immersive iRacing simulation, and (3) non-immersive FIFA console gaming. Responses during activity and immediate recovery were evaluated relative to baseline values. We hypothesized that VR table tennis would induce the greatest cardiovascular and autonomic change because it combines immersive sensory input with higher physical movement demands. We further expected that iRacing would elicit measurable responses through sustained attentional and motor-control demands, whereas FIFA would primarily reflect cognitive and emotional engagement with comparatively smaller physiological changes. Accordingly, the present comparisons should be interpreted as differences across task formats combining immersion level and physical demand, rather than as isolated effects of immersion alone. Future studies using identical tasks across different display conditions are warranted to disentangle these influences.

## 2 Methods

### 2.1 Participants

The study included a sample of 87 undergraduate students, consisting of 51 females. The participants ranged in age from 18 to 23, with a mean age of 20.5 years (SD = 1.44). We recruited participants through noticeboards, the internet, and the intranet. Participants selected their preferred group based on their prior experience and interests. Prior familiarity with each modality was determined through participant self-report during recruitment and group allocation. Participants were allocated to task groups according to prior familiarity and interest in order to minimize acute learning effects and excessive familiarization demands across substantially different modalities. However, no standardized quantitative proficiency scale was administered. The experimental tasks involved fully immersive VR table tennis (*n* = 17), semi-immersive iRacing simulation (*n* = 40), and non-immersive FIFA gaming (*n* = 30).

The selected tasks were designed to represent commonly used gaming and simulation environments differing in immersion and physical demand, rather than formal tournament-style esports competition. Participants were required to abstain from using any medications or ergogenic aids that could affect the functions of the nervous and cardiovascular systems. Additionally, they were required to have no known acute or chronic cardiovascular or psychiatric conditions. The local ethics committee approved the experimental procedures, and all participants signed informed consent forms prior to the commencement of the study. We collected the data in accordance with the most recent version of the Helsinki Declaration.

### 2.2 Measures

#### 2.2.1 HR and HRV measures

All physiological data were continuously collected throughout the experiment utilizing a portable Nexus-10 Mark II recording device (Mind Media CV; Roermond, Herten, the Netherlands) in conjunction with its software (BioTrace, MindMedia BV, the Netherlands) and the associated sensors. These sensors were interfaced with the recording computer via Bluetooth technology. HR and HRV data were acquired through electrocardiography (ECG) employing a lead II configuration with three Ag/AgCl electrodes. One electrode was positioned beneath the right clavicle, another was placed on the left side of the chest beneath the sixth rib, and the ground electrode was situated beneath the left clavicle. The ECG signals were recorded at a resolution of 24 bits and a sampling rate of 1,024 Hz, while implementing a 50 Hz notch filter to eliminate interference. Electrocardiographic (ECG) data were used to derive beat-to-beat R-R intervals for HR and HRV analyses. Signal recordings were visually inspected prior to analysis, and segments affected by excessive noise or movement artifact were corrected or excluded where necessary according to standard HRV preprocessing procedures.

#### 2.2.2 HRV analysis

We paid particular attention to artifact correction and signal loss during the experiments. We used the supplied software, BioTrace+, for the NeXus-10 device to correct the artifacts. To obtain artifact-free HRV data, we used the “set artifact area” and “automatic artifact rejection-interpolation” function of BioTrace+ software. Accordingly, we made the following selections from the “set artifact area” menu.

1-Manual and automatic Artifact Rejection option/interpolation module in BioTrace. 2-Replace IBI by interpolated data using IBI [*n*] = [IBI (*n* – 1) + IBI]/2 method. 3-Automatic removal criteria: percent difference: removal if the difference between IBI [*n*] and IBI (*n* + 1) is greater than 20%. Consequently, these artifact-free HRV data were used in the statistical analysis. HRV data were excluded from the statistical analysis when HRV artifacts exceeded 10%.

According to the recommendations set forth by the European Task Force (European Task Force, 1996), HRV analyses were conducted in both time and frequency domains using the IBI time series. The time-domain indices employed in the present study included NNmean (ms), SDNN (ms), and RMSSD (ms). For the frequency-domain analysis, we applied the fast fourier transform (FFT) algorithm, utilizing a window length of 512 and a Hanning window, resulting in a total of 1,024 points. The power spectral density was assessed for the primary frequency indices: low-frequency (LF) and high-frequency (HF) power (ms^2^). Furthermore, the relationship between these bands, expressed as the LF/HF ratio, was also subjected to analysis.

#### 2.2.3 Fully immersive setup for VR table tennis

We used the Eleven Table Tennis (For Fun Labs, USA, 2016), gaming applications for the execution of the VR table tennis, which is available in the Steam Library. The application was launched on a desktop computer equipped with an Nvidia GeForce RTX 2070 graphics card, 16 GB of RAM, and an AMD Ryzen 5 2600X six-core processor. The virtual environment was presented via an HTC Vive Pro head-mounted display (HTC Inc., Taoyuan City, Taiwan), with a resolution of 2,880 × 1,600 pixels, updated at 90 times per second, and featuring a horizontal and vertical field of view of 110°. The VR apparatus gives the impression of complete immersion in a virtual environment.

#### 2.2.4 Semi-immersive setup for iRacing

We employed an Ortombo GTR GTS Cockpit Racing Simulator (Ortombo Technology Trade Co., Ltd., Antalya, Türkiye) in conjunction with a Thrustmaster driving wheel and pedal set to facilitate the driving task simulation. For visual stimulation and the simulation-based experimental tasks, we utilized a Samsung (Samsung Electronics, Suwon, South Korea) 55-inch 4K UHD LED television (Model: UE55BU8100U) as our primary display unit. This television boasts a resolution of 3,840 × 2,160, HDR10+ support, and a 60 Hz refresh rate, ensuring high-definition clarity and seamless visual transitions.

Participants took part in a racing car driving simulation using the iRacing platform. Each participant drove a Skip Barber 2000 racing car, all with identical setups, on the Red Bull Ring—Grand Prix version of the track. We used the same desktop computer previously mentioned to deliver the driving experience.

#### 2.2.5 Non-immersive setup for FIFA

The simulation ran on a PlayStation 5 (Sony Interactive Entertainment, Tokyo, Japan) using FIFA 23 (EA Sports). Participants used the standard DualSense wireless controller in “Classic” layout. For visual stimulation, we used a Samsung (Samsung Electronics, Suwon, South Korea) 55-inch 4K UHD LED television (Model: UE55BU8100U), the same as in the iRacing task. This display features a 3,840 × 2,160 resolution, HDR10+ support, and a 60 Hz refresh rate with game mode enabled at its native resolution via HDMI, ensuring high-definition, smooth visuals. System performance mode was set to favor frame rate. Audio was delivered via closed-back headphones to standardize arousal cues. All games used Kick-Off mode with club teams. Half-length was fixed at 4 min to match the physiological recording window. Weather, stadium, and time-of-day were set to default; pitch wear and ball type were standardized. Camera angle was kept constant (e.g., Tele Broadcast with default height/zoom). Controller assists (pass, through-ball, shot, cross, and lob) and timed finishing were fixed across participants; vibration and adaptive triggers were disabled to reduce haptic variability. The opponent was a CPU team at a single, pre-set difficulty; team overall ratings were matched within ±2 points to minimize imbalance.

#### 2.2.6 Experimental procedures

**Figure 1:**
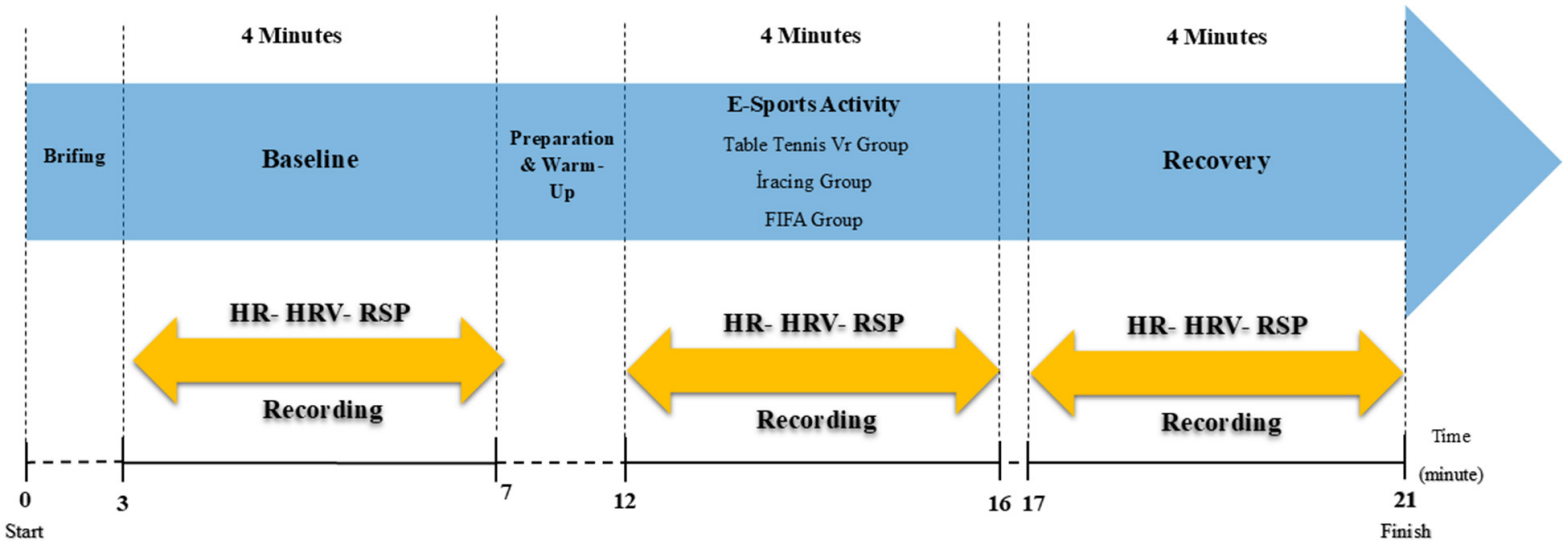
Experimental flowchart illustrating the sequential phases of the protocol. The diagram shows the timeline of the study, beginning with a briefing period, followed by a 4-min baseline phase and a preparation/warm-up period. Participants then complete a 4-min esports task (VR table tennis, iRacing, or FIFA), after which they enter a 4-min recovery phase. HR and HRV are continuously recorded during the baseline, activity, and recovery periods.

The experimental protocol followed a standardized timeline with a total duration approximately 21 min. Psychophysiological data were recorded continuously throughout the session; however, for statistical analysis, data were segmented into three 4-min epochs using time stamps, as illustrated in Figure 1.

##### 2.2.6.1 Briefing

The session commenced with a 3-min briefing on procedures. Although physiological recording was active during this period, data from this phase were excluded from analysis to prevent artifacts resulting from verbal interaction.

##### 2.2.6.2 Baseline

After the briefing, a 4-min baseline epoch was designated. Participants sat quietly without engaging in physical or cognitive tasks. Data from this interval were used to establish baseline autonomic levels. We recorded participants' resting HR and HRV activity in accordance with the recommendations of Laborde et al. (2017) and Quintana and Heathers (2014).

##### 2.2.6.3 Familiarization, warm-up, and activity

A 5-min interval was allocated for equipment setup and warm-up. Data recorded during this period were excluded from analysis to ensure standardization, as warm-up procedures (such as equipment adjustment or tactical setup) varied between groups. The main experimental task lasted 4 min. Time stamps indicated the start and end of the activity. Psychophysiological data from this window were extracted as the activity dataset.

##### 2.2.6.4 Table tennis group

Participants completed a 4-min virtual table-tennis task using the Eleven Table Tennis application. The task comprised two consecutive 2-min blocks: backhand-only strokes followed by forehand-only strokes. In the virtual environment, balls were delivered from an automated ball-feeding machine positioned on the opposite side of the participants. All participants were instructed to maintain a consistent stance and strike pace throughout the task while keeping the headset and controllers.

##### 2.2.6.5 iRacing group

Participants were allowed to complete a 2-lap time trial (approximately 5 min) for familiarization. Participants were also allowed to adjust ergonomic settings according to personal comfort, including steering wheel position, pedal reach, and seat position. Afterward, participants completed the experimental 2-lap time trial. We used the Red Bull Ring racing circuit. Participants drove a Mazda MX-5 with a manual transmission. Participants were not allowed to use the standard driving aids provided by the iRacing sim-racing platform. To ensure comparability across groups, only the first 4 min of HR and HRV recordings obtained during the experimental task were included in the analyses.

##### 2.2.6.6 FIFA group

Participants in the FIFA group were allowed to choose their preferred team from the English Premier League, La Liga, or Bundesliga. Participants then selected their preferred starting lineup and team formation. Each participant completed a standardized 5-min match against the PS5 computer opponent. To ensure comparability across experimental conditions, only the first 4 min of HR and HRV recordings during gameplay were included in the analyses, as identical 4-min activity periods were used for all groups.

##### 2.2.6.7 Recovery

Upon completion of the experimental tasks, we recorded the participants' HR and HRV recovery responses for a duration of 4 min, adhering to the procedures outlined in the baseline section. Lastly, participants were debriefed and the experiment ended.

#### 2.2.7 Statistical analysis

To analyze the dataset obtained, we initially performed a log transformation (Log 10) of the HRV frequency-domain parameters to meet the requirements for linear analysis. Subsequently, we conducted a series of repeated measures ANOVAs to evaluate whether HR and HRV responses exhibited significant differences among the baseline, activity, and recovery conditions across the experimental groups, which included VR table tennis, iRacing, and FIFA.

In the reporting of the repeated measures ANOVA, we applied a corrected degree of freedom using Greenhouse–Geisser estimates of sphericity whenever the assumption of sphericity was violated. Effect sizes were reported as partial eta-squared ($\eta_{\text{p}}^{2}$).

Upon obtaining significant results from the repeated measures ANOVA, we executed paired sample *t*-tests with a Bonferroni correction. In this context, the standard alpha level of 0.05 was divided by the number of *t*-tests conducted as *post hoc* analyses, resulting in alpha level of 0.017 for the pairwise comparisons.

Finally, we investigated whether the psychophysiological indices recorded in this study displayed significant differences between the experimental groups through a series of one-way ANOVAs. To address the considerable interindividual variability in psychophysiological parameters, particularly in HRV frequency-domain indices, we calculated percentage change for each parameter. Relative changes for each experimental period (activity and recovery) were calculated as percentage changes from baseline using the standard formula: [(period value – baseline value)/(baseline value)] × 100. We then utilized these changes in values during the activity and recovery phases as dependent variables in the one-way ANOVAs.

In light of the recommendations put forth by Laborde et al. (2017), which emphasize the significant interindividual variability observed in heart rate variability (HRV) metrics, we have determined to employ a within-subject design and analysis. This methodology allows for an initial assessment of temporal responses within the group, followed by evaluations of relative changes from baseline across different groups. This approach is intended to reduce the impact of considerable baseline variability frequently encountered in HRV indices among individuals.

## 3 Results

### 3.1 VR table tennis group

**Figure 2:**
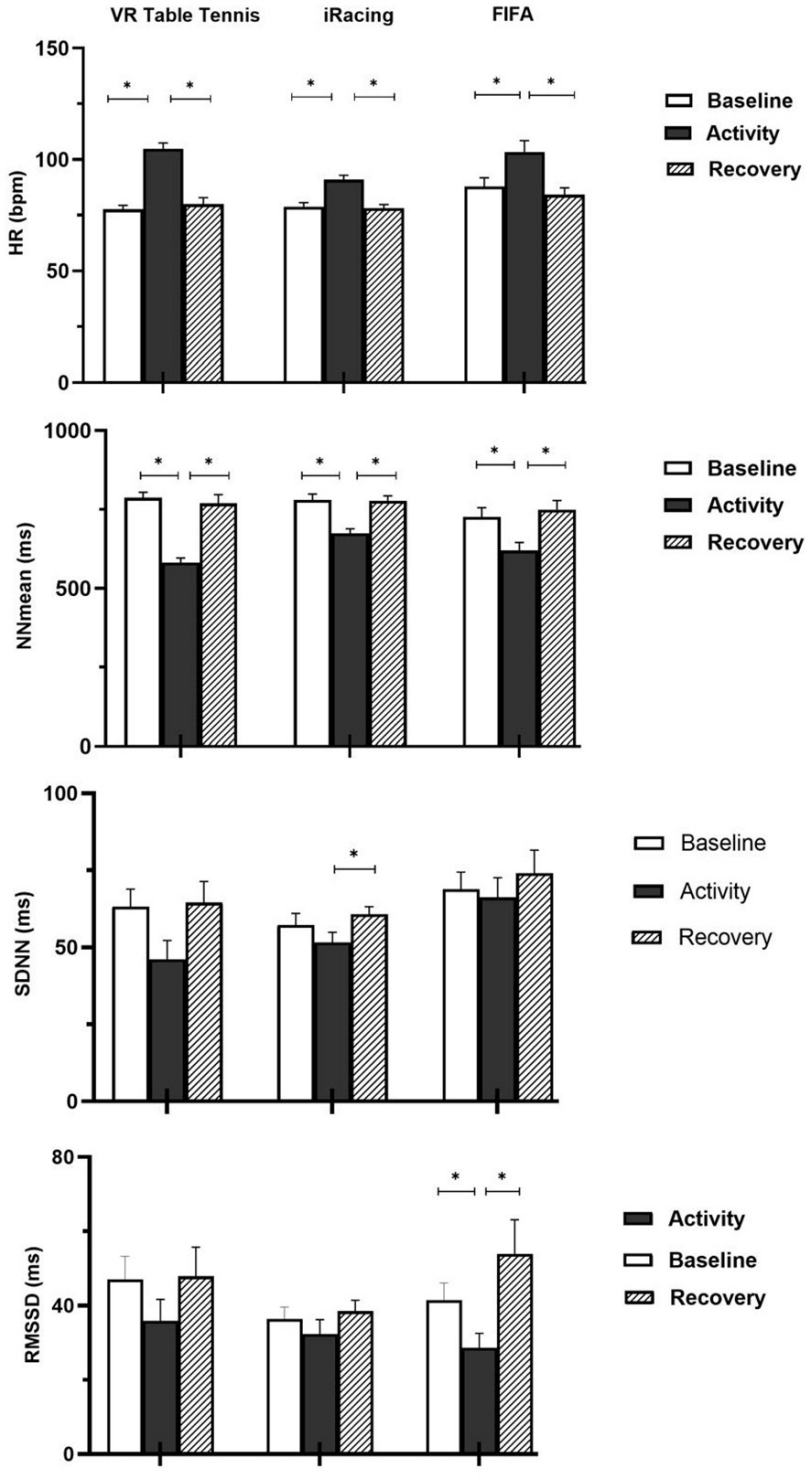
Time-domain HRV responses across VR table tennis, iRacing, and FIFA groups. HR, NNmean, SDNN, and RMSSD, changes in VR table tennis, iRacing, and FIFA groups. Note that HR increased significantly from baseline during activity and decreased to baseline during recovery in all experimental groups. NNmean decreased significantly from baseline to activity and increased significantly from activity to recovery in all experimental groups. SDNN increased significantly during recovery only in the racing group. RMSSD decreased significantly during activity and increased significantly during recovery only in the FIFA group. *p < 0.017.

**Figure 3:**
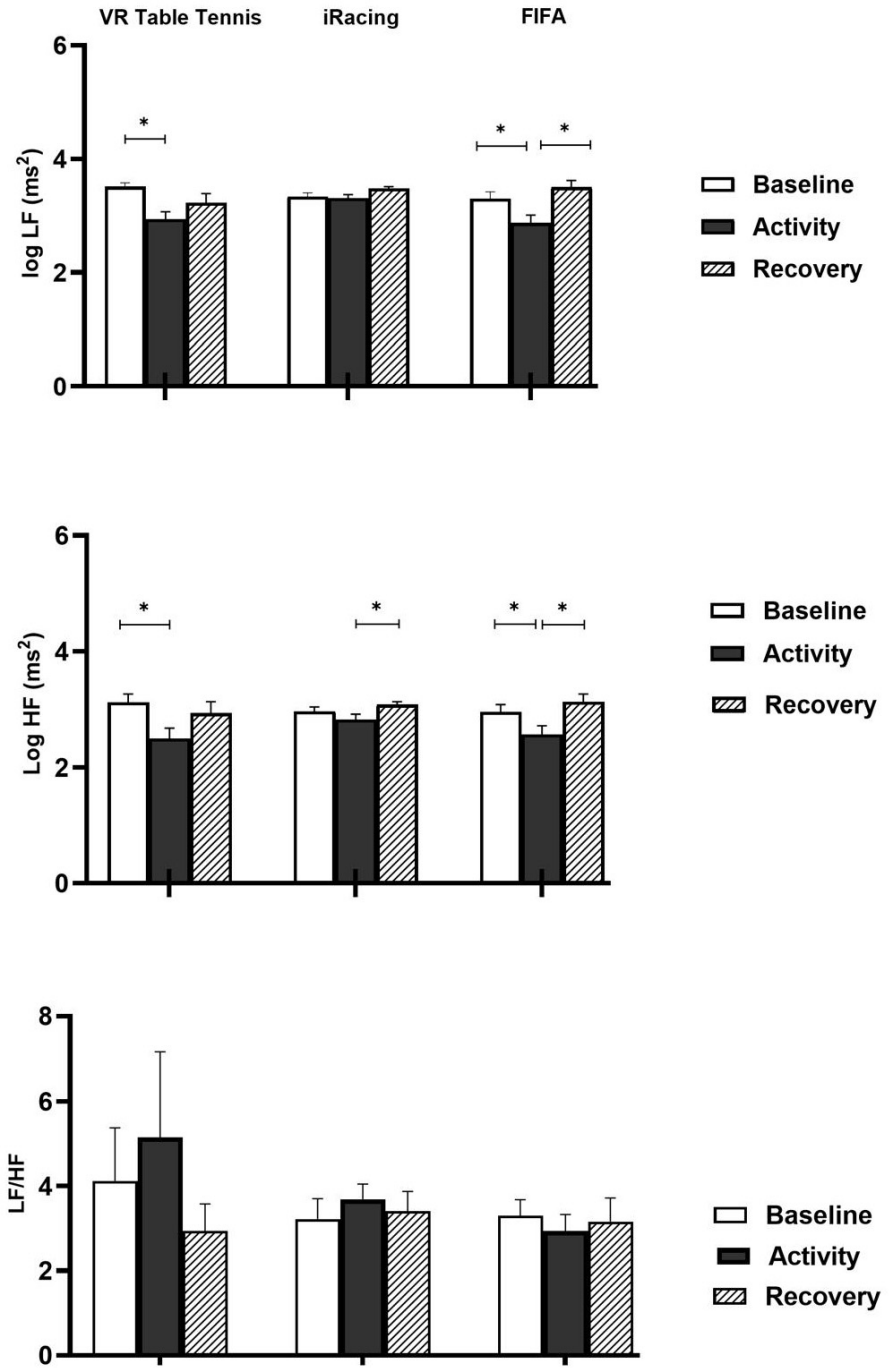
Frequency domain HRV responses across VR table tennis, iRacing, and FIFA groups. log LF, log HF, and LF/HF ratio changes in VR table tennis, iRacing, and FIFA groups. Note that log LF decreased significantly from baseline to activity in the VR table tennis. Log LF decreased from baseline to activity and increased from activity to recovery significantly in the FIFA group. Log HF decreased significantly during the activity in the VR table tennis. In the iRacing, log HF increased significantly during the recovery. Log HF decreased during the activity and increased significantly during recovery in the FIFA compared to baseline level. LF/HF ratio changes were not statistically significant for any groups. *p < 0.017.

As demonstrated in Table 1a, the results of the repeated measures of ANOVA showed that HR [*F*_(2, 32)_ = 120.45, *p* =.001, $\eta_{\text{p}}^{2}$ = 0.88], NNmean [*F*_(2, 32)_ = 93.86, *p* = 0.001, $\eta_{\text{p}}^{2}$ = 0.85], SDNN, [*F*_(2, 32)_ = 4.93, *p* = 0.014, $\eta_{\text{p}}^{2}$ = 0.23], log LF power [*F*_(1.37, 22.02)_ = 6.22, *p* = 0.013, $\eta_{\text{p}}^{2}$ = 0.28], and log HF power [*F*_(1.35, 21.74)_ = 6.94, *p* = 0.010, $\eta_{\text{p}}^{2}$ = 0.30] varied significantly among the experimental periods of baseline, activity, and recovery. On the other hand, there were no significant changes in RMSSD [*F*_(2, 32)_ = 1.85, *p* = 0.174, $\eta_{\text{p}}^{2}$ = 0.10], and LF/HF [*F*_(1.17, 18.73)_ = 1.50, *p* = 0.240, $\eta_{\text{p}}^{2}$ = 0.09] parameters. Table 1b and Figures 2, 3 illustrate the results of the follow-up multiple comparison tests as *post hoc*. Follow-up multiple comparisons with Bonferroni correction revealed that HR elevated significantly from baseline to activity in the VR table tennis group. However, HR did not vary significantly between baseline and recovery periods. HR decelerated significantly from the activity to the recovery period. NNmean decreased significantly from the baseline period to the activity period. The difference in the mean NNmean between the baseline and recovery periods was not statistically significant. However, the NNmean recorded during the recovery period was significantly lower than that observed during the activity period. Both log LF and log HF power decreased significantly from the baseline to activity periods. On the contrary, log LF and log HF power did not change significantly between baseline and recovery periods. Similarly, the differences in activity and recovery between log LF and log HF power were not statistically significant.

**Table 1a:** Results of the repeated measure of ANOVA: time and frequency domain parameters for VR table tennis group.

Variables	Baseline		Activity		Recovery		*F*	*p*	η^2^
	Mean	SD	Mean	SD	Mean	SD			
HR (bpm)	77.65	7.3	104.83	10.83	79.95	12.13	120.45	0.001^*^	0.88
NNmean (ms)	781.06	71.78	581.31	58.58	769.19	113.56	93.86	0.001^*^	0.85
SDNN (ms)	63.23	23.37	46.11	25.22	64.48	28.50	4.93	0.014^*^	0.23
RMSSD (ms)	49.97	25.90	35.85	24.06	47.8	32.4	1.85	0.174	0.10
Log LF (ms^2^)	3.51	.29	2.94	.51	3.23	.69	6.22	0.013^*^	0.28
Log HF (ms^2^)	3.12	.59	2.5	.73	2.94	.81	6.94	0.010^*^	0.30
LF/HF	4.1	5.1	5.15	8.27	2.9	2.65	1.5	0.240	0.09

^*^indicates statistically significant differences (*p* < 0.05).

**Table 1b:** Results of the multiple comparisons with Bonferroni correction for VR table tennis group.

	*t*	*p*	*t*	*p*	*t*	*p*
	a		b		c	
HR	−12.6	**0.001** ^*^	−1.2	0.222	13.44	**0.001** ^*^
NNmean	35.41	**0.001** ^*^	.727	0.478	−10.25	**0.001** ^*^
SDNN	2.55	0.021	–.244	0.810	−2.43	0.027
Log LF	4.72	**0.001** ^*^	1.99	**0.064**	−1.3	0.188
Log HF	4.25	**0.001** ^*^	1.33	0.201	−1.99	0.063
	a		b		c	
HR	−12.6	**0.001** ^*^	−1.2	0.222	13.44	**0.001** ^*^
NNmean	35.41	**0.001** ^*^	0.727	0.478	−10.25	**0.001** ^*^
SDNN	2.55	0.021	−0.244	0.810	−2.43	0.027
Log LF	4.72	**0.001** ^*^	1.99	**0.064**	−1.3	0.188
Log HF	4.25	**0.001** ^*^	1.33	0.201	−1.99	0.063
	a		b		c	
HR	−12.6	**0.001** ^*^	−1.2	0.222	13.44	**0.001** ^*^
NNmean	35.41	**0.001** ^*^	0.727	0.478	−10.25	**0.001** ^*^
SDNN	2.55	0.021	−0.244	0.810	−2.43	0.027
Log LF	4.72	**0.001** ^*^	1.99	**0.064**	−1.3	0.188
Log HF	4.25	**0.001** ^*^	1.33	0.201	−1.99	0.063

a, comparison between baseline and activity conditions; b, comparison between baseline and recovery; c, comparison between activity and recovery. Bold values indicate statistically significant results. ^*^indicates statistically significant differences (*p* < 0.05).

### 3.2 iRacing group

Table 2a, Figures 2, 3 demonstrate the mean differences of HR and HRV in the iRacing group. Hence, the results of the repeated measures of ANOVA indicated that HR [*F*_(1.64, 65.83)_ = 65.89, *p* = 0.001, $\eta_{\text{p}}^{2}$ = 0.62], NNmean [*F*_(1.72, 68.84)_ = 62.23, *p* = 0.001, $\eta_{\text{p}}^{2}$ = 0.61], SDNN, [*F*_(1.66, 66.39)_ = 4.70, *p* = 0.017, $\eta_{\text{p}}^{2}$ = 0.10], and log HF power [*F*_(1.42, 56.93)_ = 5.25, *p* = 0.015, $\eta_{\text{p}}^{2}$ = 0.12] varied significantly among the baseline, activity, and recovery periods. Conversely, the changes observed in RMSSD [*F*_(1.34, 53.95)_ = 2.01, *p* = 0.157, $\eta_{\text{p}}^{2}$ = 0.04], log LF [*F*_(1.53, 61.37)_ = 3.10, *p* = 0.065, $\eta_{\text{p}}^{2}$ = 0.07], and LF/HF ratio [*F*_(1.67, 67.14)_ = 0.547, *p* = 0.551, $\eta_{\text{p}}^{2}$ = 0.01] were not statistically significant. As illustrated in Table 2b, multiple comparisons with Bonferroni correction revealed that HR increased significantly from baseline to activity. The baseline and recovery difference in HR was not statistically significant. Mean HR during recovery was significantly lower than the mean HR during activity. NNmean decreased significantly from baseline to activity. Mean NNmean difference between baseline and recovery was not statistically significant. However, NNmean elevated significantly from the activity to the recovery period. SDNN difference between baseline and activity was not statistically significant. Similarly, baseline recovery difference in SDNN was not statistically significant. However, SDNN increased significantly from the activity to the recovery period.

**Table 2a:** Results of the repeated measure of ANOVA: time and frequency domain parameters for iRacing group.

Variables	Baseline		Activity		Recovery		*F*	*p*	η^2^
	Mean	SD	Mean	SD	Mean	SD			
HR (bpm)	87.87	11.16	90.95	12.75	78.20	10.3	65.89	0.001^*^	0.62
NNmean (ms)	780.35	117.04	673.82	95.12	777.27	102.78	62.23	0.001^*^	0.61
SDNN (ms)	57.21	24.54	51.63	20.63	60.83	15.24	4.7	0.017^*^	0.10
RMSSD (ms)	36.32	21.15	32.30	25.08	38.45	18.92	2.01	0.157	0.04
Log LF (ms^2^)	3.34	.35	3.31	.41	3.48	.25	3.10	0.065	0.07
Log HF (ms^2^)	2.97	.42	2.83	.58	3.08	.38	5.25	0.015^*^	0.12
LF/HF	3.22	3.06	3.69	2.3	3.41	2.9	.547	0.551	0.01

^*^indicates statistically significant differences (*p* < 0.05).

**Table 2b:** Multiple comparisons with Bonferroni correction for iRacing group.

	a		b		c	
	*t*	*p*	*t*	*p*	*t*	*p*
HR	−8.4	0.001^*^	0.723	0.474	9.61	0.001^*^
Nnmean	8.5	0.001^*^	0.355	0.725	−9.35	0.001^*^
SDNN	1.3	0.133	−1.4	0.170	−3.37	0.002^*^
Log HF	1.6	0.117	−2.33	0.024	−2.79	0.008^*^

a, comparison between baseline and activity conditions; b, comparison between baseline and recovery; c, comparison between activity and recovery. ^*^indicates statistically significant differences (*p* < 0.05).

### 3.3 FIFA group

Table 3a, Figures 2, 3 illustrate the mean differences of HR and HRV indices in the FIFA group. Accordingly, the results of the repeated measures of ANOVA indicated that HR [*F*_(2, 58)_ = 26.82, *p* = 0.001, $\eta_{\text{p}}^{2}$ = 0.48], NNmean [*F*_(1.20, 34.83)_ = 10.08, *p* = 0.002, $\eta_{\text{p}}^{2}$ = 0.26], RMSSD, [*F*_(1.23, 35.78)_ = 6.27, *p* = 0.012, $\eta_{\text{p}}^{2}$ = 0.18], log LF power [*F*_(2, 58)_ = 8.23, *p* = 0.001, $\eta_{\text{p}}^{2}$ = 0.22], and log HF power [*F*_(2, 58)_ = 10.05, *p* = 0.001, $\eta_{\text{p}}^{2}$ = 0.26] changed significantly among the experimental periods of baseline, activity, and recovery. By contrast, analyses indicated no significant changes for SDNN [*F*_(2, 58)_ = 2.44, *p* = 0.096, $\eta_{\text{p}}^{2}$ = 0.08], LF/HF ratio [*F*_(1.45, 42.06)_ = 0.79, *p* = 0.422, $\eta_{\text{p}}^{2}$ = 0.03] parameters. As shown in Table 3b, HR elevated significantly from the baseline to the activity period. The HR difference between baseline and recovery was not significant. The HR during the recovery period was significantly lower compared to the HR during the baseline period. The HRV time-domain parameter of NNmean decreased significantly from baseline to activity. There was no significant difference in NNmean between the baseline and recovery periods in the FIFA group. However, NNmean during the recovery period was significantly higher compared to NNmean during the activity. There was a significant decrease in RMSSD from the baseline to the activity period. In the FIFA group, RMSSD did not change between the baseline and recovery periods. However, the RMSSD recorded during the recovery period was significantly higher than the RMSSD recorded at activity. Both log LF and log HF power decreases from baseline to activity were statistically significant. The log LF and log HF power differences between baseline and recovery did not reach a significance level. Conversely, the log LF and log HF power recorded during recovery were significantly greater than the log LF and log HF power recorded during activity.

**Table 3a:** Results of the repeated measure of ANOVA: time and frequency domain parameters for FIFA group.

Variables	Baseline		Activity		Recovery		*F*	*p*	η^2^
	Mean	SD	Mean	SD	Mean	SD			
HR (bpm)	87.88	21.45	103.46	27.5	84.18	17.21	26.82	0.001^*^	0.48
NNmean (ms)	724.12	161.85	619.6	141.63	749.47	155.85	10.08	0.002^*^	0.26
SDNN (ms)	68.91	30.38	66.19	35.29	74.01	41.44	2.44	0.096	0.08
RMSSD (ms)	41.42	25.73	28.66	21.22	53.87	50.62	6.27	0.012^*^	0.18
Log LF (ms^2^)	3.30	0.67	2.87	0.78	3.5	0.70	8.23	0.001^*^	0.22
Log HF (ms^2^)	2.96	0.63	2.57	0.80	3.13	0.72	10.05	0.001^*^	0.26
LF/HF	3.3	2.1	2.94	2.11	3.16	3.11	0.792	0.422	0.03

^*^indicates statistically significant differences (*p* < 0.05).

**Table 3b:** Multiple comparisons with Bonferroni correction for FIFA group.

	a		b		c	
	*t*	*p*	*t*	*p*	*t*	*p*
HR	−5.19	0.001^*^	1.73	0.094	6.14	0.001^*^
NNmean	2.85	0.008^*^	−1.92	0.064	−3.6	0.001^*^
RMSSD	3.76	0.001^*^	−1.56	0.129	−2.87	0.008^*^
Log LF	2.59	0.015^*^	−1.25	0.218	−4.09	0.001^*^
Log HF	3.12	0.004^*^	−1.21	0.237	−4.05	0.001^*^

a, comparison between baseline and activity conditions; b, comparison between baseline and recovery; c, comparison between activity and recovery. ^*^indicates statistically significant differences (*p* < 0.05).

**Figure 4:**
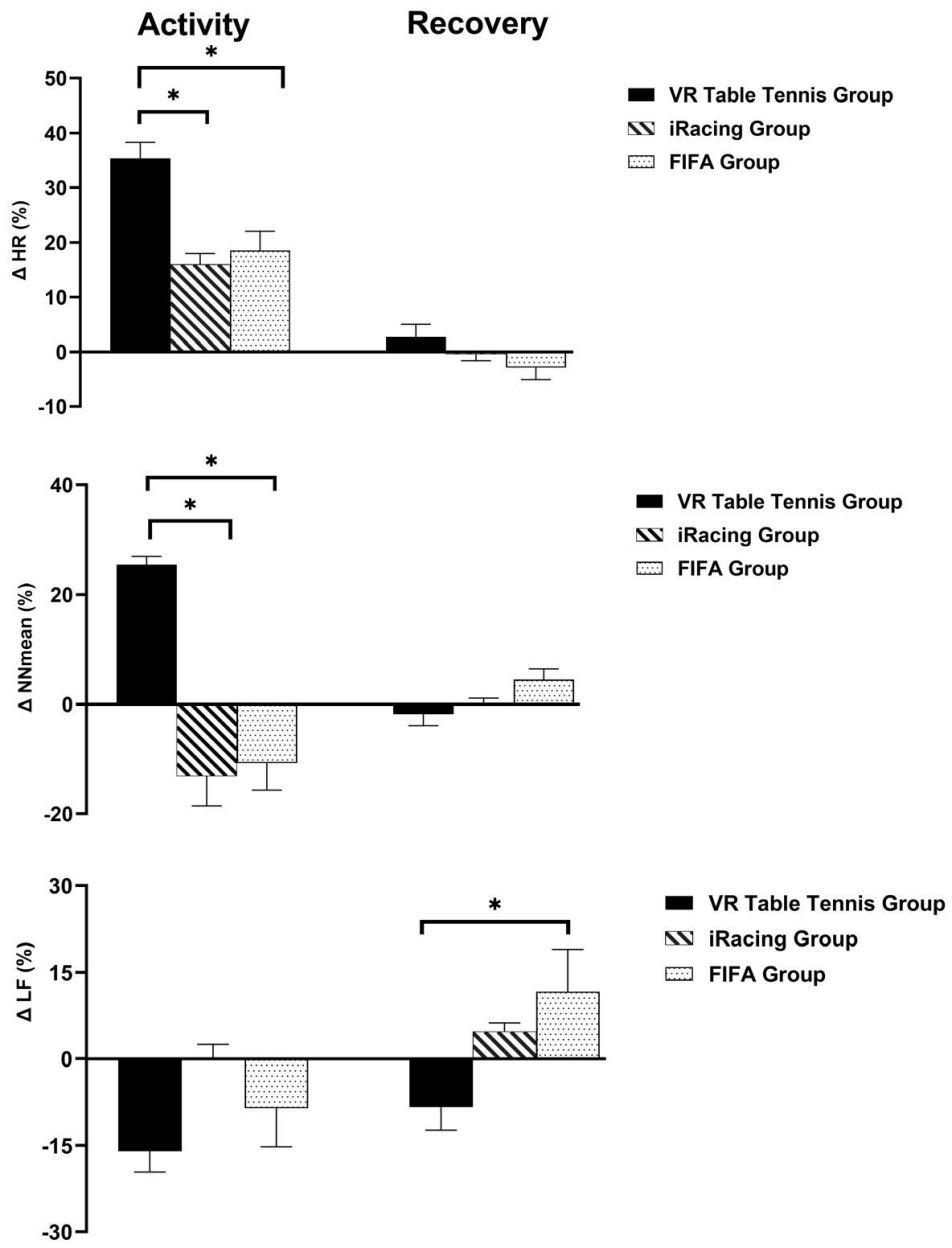
Percentage changes in HR, NNmean, and log LF among VR table tennis, iRacing, and FIFA groups. Comparison of the percentage changes in HR, NNmean, and log LF among experimental groups. During the activity, immersive table tennis induced a significantly greater increase in HR than iRacing and FIFA. VR table tennis elicited greater NNmean decrease than iRacing and FIFA during the activity. Although the one-way ANOVA revealed a significant main effect, multiple comparisons did not detect any significant differences in NNmean among the experimental groups during recovery. Changes in log LF did not differ significantly among the experimental groups during activity. During the recovery, FIFA led to higher log LF increase than VR table tennis. *indicates statistically significant differences between groups (*p* < 0.05).

We conducted a series of one-way ANOVAs followed by Tukey *post hoc* tests to investigate whether the percentage change in HR and HRV during activity and recovery varied significantly among VR table tennis, iRacing, and FIFA. As presented in Table 4, the findings indicated a significant percentage change in HR during activity. Notably, VR table tennis resulted in a more substantial percentage change in HR compared to iRacing and FIFA. As demonstrated in Figure 4, descriptive statistics revealed that VR table tennis led to a 35.7% increase in HR, whereas iRacing and FIFA showed increases of only 18.54% and 16.01%, respectively. The percentage change in the HRV time-domain index of NNmean also varied significantly during the activity. Accordingly, VR table tennis led to a greater decrease in NNmean than iRacing and FIFA. Descriptive statistics demonstrated that VR table tennis decreased NNmean by 25.37%, while iRacing and FIFA decreased NNmean by only 13.10% and 10.73%, respectively. One-way ANOVA indicated a significant difference in NNmean during recovery. However, as illustrated in Figure 4, *post hoc* Tukey tests did not reveal any significant difference in the percentage change of NNmean during the recovery. Lastly, the percentage change of LF power varied significantly during recovery. In this respect, VR table tennis resulted in a greater percentage change in LF power compared to FIFA.

**Table 4:** One-way ANOVA of change scores of HR and HRV indices during the activity and recovery among the experimental groups.

Variables	Change scores	Groups	Mean	SD	*F*	*p*
HR	Activity	VR TT	35.37	12.04	10.023	<0.001*
		FIFA	18.54	19.54		
		iRacing	16.01	12.78		
	Recovery	VR TT	2.69	9.57	1.76	0.178
		FIFA	–2.85	12.24		
		iRacing	–0.40	7.67		
NNmean	Activity	VR TT	–25.37	6.42	4.225	0.018*
		FIFA	–10.73	27.15		
		iRacing	–13.10	8.95		
	Recovery	VR TT	–1.78	8.59	3.49	0.035*
		FIFA	4.45	10.82		
		iRacing	0.06	6.68		
SDNN	Activity	VR TT	–23.40	35.21	2.010	0.140
		FIFA	–11.65	36.09		
		iRacing	-–31.64	35.08		
		VR TT	2.26	33.28	0.50	0.610
		FIFA	15.75	68.69		
		iRacing	13.96	28.33		
RMSSD	Activity	VR TT	-–10.75	62.97	1.802	0.171
		FIFA	–27.46	36.78		
		iRacing	–3.75	57.01		
	Recovery	VR TT	0.30	33.06	2.11	0.128
		FIFA	49.48	144.85		
		iRacing	13.74	33.64		
LF	Activity	VR TT	–16.00	14.93	2.771	0.068
		FIFA	–8.54	37.04		
		iRacing	0.13	15.26		
	Recovery	VR TT	–8.32	16.50	3.35	0.040
		FIFA	11.64	40.23		
		iRacing	4.72	9.61		
HF	Activity	VR TT	–19.63	19.29	0.220	0.139
		FIFA	–11.12	39.44		
		iRacing	–3.96	23.92		
	Recovery	VR TT	–5.94	17.07	1.92	0.153
		FIFA	9.16	39.67		
		iRacing	4.41	10.98		
LF/HF	Activity	VR TT	55.17	122.31	2.980	0.056
		FIFA	9.78	67.45		
		iRacing	72.02	122.78		
	Recovery	VR TT	33.23	135.33	1.72	0.186
		FIFA	–2.85	244.84		
		iRacing	–21.20	127.16		

*indicates statistically significant differences (p < 0.05).

## 4 Discussion

The primary aim of the present study was to compare short-term autonomic responses across digital gaming and simulation tasks differing in immersion level and physical demand. The findings demonstrated that all three modalities elicited significant increases in HR together with reductions in HRV from baseline to activity, indicating measurable autonomic activation during task engagement. However, the magnitude of these responses appeared to be more strongly influenced by physical demand than immersion level alone, as the fully immersive VR table tennis condition produced the greatest cardiovascular change. Overall, these findings suggest that gaming and simulation tasks may induce psychophysiological responses above resting levels, although task-specific movement and workload characteristics appear to be more influential than immersion status by itself.

The observed increase in HR together with reductions in HRV during task performance is consistent with previous literature showing that cognitively engaging or movement-based digital tasks can produce acute autonomic activation. Short-term decreases in vagally mediated HRV are commonly interpreted as reflecting increased regulatory demand, attentional engagement, or effort mobilization during active task performance. Similar responses have been reported during interactive virtual environments and exergaming tasks in which users must continuously respond to changing stimuli and maintain performance under time constraints (Aubert et al., 2003; Thayer and Lane, 2000; Rutkowski et al., 2021; Ahmed et al., 2020). Therefore, the larger responses observed in the VR table tennis condition cannot be attributed solely to immersive technology, but likely reflect the combined effects of greater whole-body movement, perceptual engagement, and task workload.

An important finding of the present study was that higher immersion did not automatically translate into larger autonomic responses across all conditions. Instead, the largest changes were observed in the VR table tennis task, which also required the greatest whole-body movement demand. This suggests that immersion may interact with physical effort, task complexity, and attentional load rather than acting as an independent determinant of HRV responses. Previous research in virtual environments has similarly suggested that presence alone does not fully explain physiological activation unless accompanied by meaningful behavioral engagement or workload demands.

The present findings indicate that engagement in esports tasks with varying levels of physical demand, whether through immersive or non-immersive technologies, can trigger autonomic adjustments, specifically reflected in HRV. Previous research exploring the relationship between VR-based esports engagement and HRV focused primarily on aerobic exercise modalities, such as cycling or moderate-intensity physical activities. In this regard, Rutkowski et al. (2021) conducted a study to evaluate individuals' performance during a submaximal incremental test under both VR and non-VR conditions. The results demonstrated that participants were able to engage in prolonged exercise durations, exhibited a lower HR, and achieved a greater RMSSD in the VR condition as compared to the non-VR scenario. Additionally, Ahmed et al. (2020) demonstrated that HRV measured during virtual exercise was comparable to that during actual cycling in participants who engaged in moderate exercise. While previous studies have provided evidence that VR can provoke HRV responses during athletic tasks that utilize large muscle groups and require aerobic endurance, there has been no investigation to date concerning whether skill and precision-based activities, such as table tennis, car driving simulation, and console soccer games with varying levels of immersion and activity, can elicit similar HRV responses. One possible explanation for these contrasting findings is that aerobic VR exercise may reduce perceived exertion through distraction and enhanced tolerance, whereas precision-skill VR tasks may increase autonomic activation through rapid movement demands, timing pressure, sensorimotor coordination, and visual-vestibular load. This unexplored area warrants thorough examination and analysis. Accordingly, the present findings should be interpreted within controlled student-based gaming and simulation conditions rather than as direct evidence for professional competitive esports or specialist training contexts.

The findings of the current study suggest that, in addition to traditional exercises simulated through VR, activities such as VR table tennis, iRacing, and FIFA also stimulate HRV responses. The Neurovisceral Integration Model, developed by Thayer and Lane (2000), offers a robust theoretical framework that can effectively elucidate the observed results. Accordingly, increased HR and reduced HRV response patterns indicate the reductions in vagally mediated HRV alongside the activity phases, in addition to partial recovery during the recovery, indicating transient down-regulation of parasympathetic vagal brake to support goal-directed control by the CAN.

These activities require attentional focus and motor control, which may influence autonomic and stress-related pathways. The presence of comparable short-term responses across different task formats suggests that simulation-based platforms may provide physiologically relevant supplementary environments for training and rehabilitation. When combined with appropriate measurement procedures and validated indicators of recovery, such platforms may also support future clinical and performance applications.

In conclusion, rather than directly replicating real-world sports, the data support the interpretation that VR and screen-based simulations impose an incremental psychophysiological load above baseline metabolic levels, thereby providing a physiologically meaningful surrogate for real-world demands.

An essential issue to examine is the variation in HR and HRV percentages during both physical activity and recovery. As shown in Figure 4, VR table tennis produced a significantly higher percentage change in HR than iRacing and FIFA during the activity phase. Among the HRV time-domain indices, only NNmean showed a significant difference. VR table tennis produced a significantly greater percentage change in NNmean than iRacing and FIFA during the activity phase. There was no significant percentage change in HRV frequency-domain indices, except LF power. In this respect, VR table tennis produced a significantly lower percentage change in LF power than iRacing and FIFA. Taken together, the findings suggest that the magnitudes of percentage changes relative to baseline across various levels of immersion and activity were limited, except for HR, NNmean, and log LF power. In other words, the cumulative load across various esports modalities was comparable despite differences in immersion levels and activity types. Consequently, despite differences in immersion and physical demand, similar percentage changes across esports modalities suggested that the influence of these factors on HRV is complex. A further consideration is that only the percentage changes in HR and NNmean observed during activity, both of which are closely associated with each other, differed significantly across esports modalities. These findings suggest that HR is more sensitive than HRV to physical and perceptual loads. Accordingly, between-group differences in change patterns should be interpreted as descriptive comparative findings rather than definitive interaction effects.

Considering these results, we can infer that VR table tennis activates large muscle groups and entails significant physical exertion. Consequently, this activity results in an elevated HR and reduced HRV to meet the metabolic demands of the engaged muscles. This phenomenon is linked to the exercise pressor effect, a vital feedback mechanism that regulates cardiovascular and respiratory responses to exercise through mechano-sensitive and metabosensitive skeletal muscle afferents (Mitchell et al., 1983).

Visual–vestibular conflict (VVC) may be a contributing factor that explains the results of the current study. VVC arises when the intense optical flow generated by head-tracked head-mounted displays (HMDs) does not match the vestibular system's input. Additional factors contributing to VVC include delayed feedback from head and trunk movements, vast fields of view, and rapid scene transitions. This multisensory mismatch can place a significant extra load on perceptual reweighting, leading to a range of responses, including motion sickness, nausea, dizziness, and autonomic discomfort (Himi et al., 2004; Ohyama et al., 2007; Malińska et al., 2015).

Within this framework, VVC elevates the integration demands on the anterior insula and anterior cingulate cortex, thereby amplifying perceived postural threat and arousal. This modulation influences the outputs of the CAN to hypothalamic and brainstem centers, such as the nucleus tractus solitarius (NTS), nucleus ambiguus, and ventrolateral medulla. As a result, there is a transient release of the vagal brake, leading to increased HR and decreased vagally mediated HR variability, as measured by RMSSD and HF components (Aubert et al., 2003; Berntson et al., 2005; Appelhans and Luecken, 2006).

While the reduced gross motor activity in iRacing might imply lower metabolic demand compared to VR Table Tennis, the sustained isometric contractions involved in steering and braking may nonetheless provoke increased cardiac output and vagal withdrawal (i.e., higher HR and lower HRV). Moreover, driving simulation also requires the use of cognitive skills, such as reaction time, spatial awareness, and concentration. This combination of physical and cognitive skills may elicit variation in ANS activity via central command. It is also possible that participants with greater familiarity or skill efficiency experienced lower perceived workload during task execution.

We anticipated that there would be no autonomic changes in the non-immersive FIFA condition. Nevertheless, the significant HR and HRV responses suggest that cognitive load, emotional arousal, and competitive engagement can stimulate the ANS even in the absence of physical movement (Arakaki et al., 2023). Thus, the findings associated with the FIFA group may reflect cognitive and emotional demands in addition to low-level physical engagement, although similar forms of attentional and cognitive demand were likely present across all task conditions. Console-based soccer games, such as FIFA, may elicit both emotional arousal and cognitive engagement as players strategies, compete, and anticipate. It is also possible that the broader attentional and tactical demands of FIFA contributed to autonomic responses, indicating that cognitive workload may represent an important mechanism beyond physical activity alone.

### 4.1 Limitations

A principal limitation of the present study is that several design and analytical constraints should be considered when interpreting causal differences across modalities. A principal limitation of the present design is that immersion level and physical task demand were inherently coupled across conditions. As a result, the independent contribution of immersion cannot be isolated from movement-related physiological load.

Although racing simulators (even without one-to-one g-force reproduction) and VR table tennis can employ suitably designed controllers, critical deficits may persist in FIFA gaming modalities, particularly in kinematic fidelity, implement mass/inertia, and resistance profiles. In passive esports such as e-football, there is no intention to replicate football-specific actions physically for the user. Researchers, coaches, and athletes should carefully and comprehensively evaluate these capabilities and limitations.

The unequal group sizes reflect the differing accessibility and appeal of each modality. Fewer participants were eligible for the physically demanding VR table tennis condition, more for the widely played FIFA game, and an intermediate number for the iRacing simulation. These differences may have influenced statistical power and should be considered when interpreting between-group comparisons. The between-group design and convenience sampling procedure may have increased inter-individual variability and limited causal comparisons across modalities. Future studies using randomized within-subject crossover designs are warranted. However, no standardized quantitative assessment of prior gaming experience, task proficiency, or cumulative exposure history was conducted. Therefore, differences in familiarity or skill level may have contributed to between-group variation in autonomic responses. In addition, the statistical approach did not include a formal mixed-design Group × Phase interaction model. Therefore, differential response trajectories across modalities should be interpreted cautiously.

The esports activities in this study required distinct preparation, warm-up, and familiarization procedures, as well as varying physical activity intensities, which may have influenced the results. Readers are therefore encouraged to consider these factors when interpreting the findings.

The current study did not address how respiration influences vagally mediated HRV. Instead, our focus was on the primary HRV parameters. Nonetheless, controlling for the effects of respiration may prove advantageous for future research. Future studies should consider the potential impact of respiration on HRV. A further limitation of this research lies in the absence of systematically collected subjective states known to influence autonomic responses and recovery processes. Factors such as perceived exertion (e.g., assessed with the Borg 6–20 or CR10 scales), perceived task difficulty, and mental workload (e.g., measured using NASA-TLX) offer valuable insights into effort and resource demands associated with vagal withdrawal. The present study did not directly quantify cognitive workload across tasks; therefore, the relative contribution of mental demand to HR and HRV responses remains uncertain.

Additionally, aspects like competitive pressure, challenge-threshold appraisal (e.g., evaluations of demands vs. resources), immersion/presence (e.g., evaluated through the IPQ or Presence Questionnaire), flow (e.g., captured by the Flow Short Scale), and sense of agency all measure experiential dimensions (e.g., control, stakes, and absorption) that could distinguish VR tasks from screen-based tasks beyond mere device differences.

Taken together, uncontrolled respiration and unmeasured subjective states may have contributed to unexplained variance in HRV and limit the precision with which specific mechanisms (e.g., metabolic vs. cognitive load) can be inferred.

### 4.2 Conclusion

The present findings indicate that the examined gaming and simulation tasks elicited measurable autonomic responses above resting baseline levels. The magnitude of these responses appeared to vary according to task-specific physical and perceptual demands. These findings support the potential value of such platforms as supplementary research and training environments, although their validity, reliability, and real-world transferability require further investigation.

### 4.3 Practical implications

The practical implications of these findings are that simulation-based tasks may become more physiologically meaningful when they reproduce key movement characteristics and mechanical demands of the target skill. In addition, tasks with relatively low metabolic demand but meaningful perceptual-motor requirements may still elicit measurable autonomic activation.

The present findings may assist researchers in understanding how immersive and non-immersive simulation tasks influence short-term autonomic responses. Simulation-based tasks may offer controlled environments for studying psychophysiological responses and perceptual-motor engagement under standardized conditions. Their applied value for specialist professional populations should be examined in task-specific future studies.

## Data Availability

The raw data supporting the conclusions of this article will be made available by the authors, without undue reservation.
